# Pancreatic Cancer-Induced Neutrophil Extracellular Traps: A Potential Contributor to Cancer-Associated Thrombosis

**DOI:** 10.3390/ijms18030487

**Published:** 2017-02-24

**Authors:** Norbaini Abdol Razak, Omar Elaskalani, Pat Metharom

**Affiliations:** 1 School of Biomedical Sciences, Curtin University, 6102 Perth, Australia; n.abdolrazak@student.curtin.edu.au; 2 Curtin Health and Innovation Research Institute, Faculty of Health Sciences, Curtin University, 6102 Perth, Australia; omar.elaskalani@postgrad.curtin.edu.au

**Keywords:** neutrophil extracellular traps, pancreatic cancer, platelets, venous thromboembolism

## Abstract

Pancreatic cancer (PaCa) is a highly metastatic cancer, and patients are at high risk of developing venous thromboembolism (VTE). Neutrophil extracellular traps (NETs) have been associated with cancer metastasis and cancer-associated thrombosis, but the ability of cancer to stimulate NET release is not known. The release of NETs has been shown to be a slow process and requires reactive oxygen species (ROS) production. Studies suggest that activated platelets are important mediators in the release. Here, we show that PaCa cells can stimulate the rapid release of NETs, independently of ROS production. We further assessed the role of platelets in PaCa-induced NETs and observed a trend of increased the NET release by PaCa-primed platelets. Additionally, NETs promoted thrombus formation under venous shear stress ex vivo. Taken together, our results suggest that PaCa-induced NETs can contribute to the high risk of venous thromboembolism development in PaCa patients, and reveal NETs as a potential therapeutic target.

## 1. Introduction

Neutrophil extracellular traps (NETs) are web-like structures composed of DNA and proteins that are expelled from activated polymorphonuclear neutrophils (PMNs) in response to bacterial or inflammatory stimuli [1,2]. They were first discovered to serve a protective role in immune defence as they can entrap pathogens, and directly kill bacteria via NET-bound antimicrobial proteins [2]. However, studies have revealed that NETs are not exclusive to bacterial infection, but are also involved in inflammatory and autoimmune diseases such as systemic lupus erythematosus, preeclampsia, rheumatoid arthritis, cystic fibrosis and thrombosis [3,4,5,6].

More recently, several links between NETs and cancer have been made. The first study investigating NETs and their association with cancer established that PMNs isolated from tumour-bearing mice are sensitised to form NETs [7]. Furthermore, spontaneous thrombi were found in association with NETs in tumour-bearing mice, suggesting a possible cause of cancer-associated thrombosis [7]. NETs have also been implicated in cancer progression, where cancer cells were found entrapped in the vasculature by NETs, which correlated with increased tumour metastases [8]. Not only have NETs been found in tumour-bearing mice [7,9], but also in tumour samples from cancer patients [9,10,11]. Despite the association between NETs and cancer, whether or not cancer cells can directly stimulate the release of NETs has not been investigated.

The molecular mechanisms of NET generation are poorly understood; however, several studies suggest that neutrophil–platelet interactions are important. Activated platelets in inflammation or infection play a vital role in the release of NETs, either by mediating interactions with neutrophils [12,13,14,15] or by soluble mediators released from platelets [16]. Platelets are also known to become activated and aggregate in cancers and are usually present in high numbers in cancer patients [17]. Thus platelets may also have a major role in NET release in cancer.

Activated platelets in contact with tumour cells can also recruit neutrophils by secreting chemokines (C-X-C motif) ligand (CXCL) 2 and CXCL5 chemokines [18]. Platelets are crucial in recruiting neutrophils in vivo as depleting platelets resulted in complete inhibition of granulocyte recruitment [18]. Thus, there is evidence to support platelets and their role in recruiting neutrophils to tumour sites; however little is known about platelets and their role in activating neutrophils in cancers [19]. Similarly, platelets have been demonstrated to interact with neutrophils and induce NET generation in bacterial infection, though in the presence of cancer, the importance of platelets in NET release has not been studied.

Therefore, given the high incidence and morbidity due to thrombotic diseases and metastasis in pancreatic cancer patients [20,21], this study aimed to investigate the interactions between neutrophils, platelets and pancreatic cancer cells and their potential contribution to the hypercoagulable state that is present with cancer-associated thrombosis. The impact of platelets and cancer cells on NETs generated, as well as the consequences of NETs on platelet activity, were investigated. We report that pancreatic cancer cells can stimulate the release of NETs, and that this is not dependent on neutrophil–cancer cell interactions as the protein fraction of cancer cell-conditioned media also induced NET release. Furthermore, we also show that NETs cause platelet activation and spreading, as well as thrombus formation under venous flow conditions. This improved understanding of these interactions may help explain contributors to the high risk of thrombosis in pancreatic cancer, and NETs or unique signalling events between cells that promote the NET release may serve as novel therapeutical targets.

## 2. Results

### 2.1. Pancreatic Cancer Cell Line AsPC-1 Stimulates NET Generation

**Figure 1:**
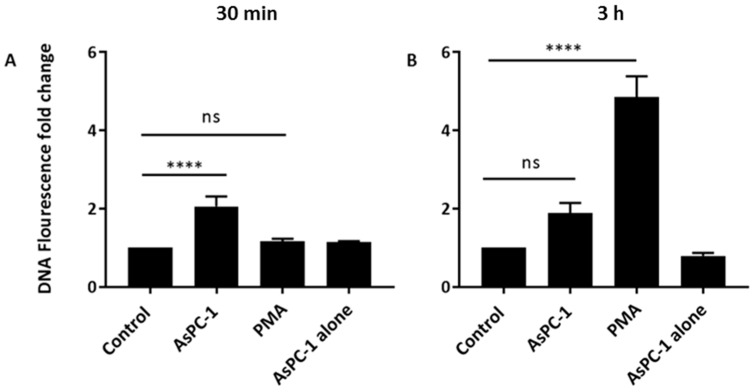
AsPC-1 stimulates the rapid release of neutrophil extracellular traps (NETs) from human neutrophils. Human neutrophils were incubated with AsPC-1 cells at a neutrophil to AsPC-1 ratio of 1:2 and NETs were quantified using Sytox Green (5 μM) in a fluorescence plate reader at (**A**) 30 min and (**B**) 3 h. AsPC-1 induced rapid NET release at 30 min while phorbyl myristate acetate (PMA)-stimulated neutrophils (positive control) required 3 h. Control: unstimulated neutrophils. (*n* = 12, **** *p* < 0.0001 and ns = non-significant); One-way ANOVA followed by Bonferroni post-test. Data presented as mean ± SEM.

To determine whether cancer cells can directly stimulate the release of NETs, pancreatic cancer cell and PMN co-cultures were performed. The metastatic pancreatic cancer cell line AsPC-1 was incubated with PMNs for 3 h, and NET release was quantified. To quantify NETs, extracellular DNA (exDNA) was stained with Sytox Green, a cell-impermeable fluorescent dye specific for DNA, and fluorescence was measured in a plate reader. NETs were quantified at 30 min, and at 3 h, as shown in Figure 1. AsPC-1 cells efficiently stimulated the release of NETs after incubation with human PMNs. In addition, NETs were induced by AsPC-1 in a rapid process as a significant 2-fold increase in exDNA release was observed at 30 min. Positive control, phorbyl myristate acetate (PMA)-stimulated PMNs required 3 h for significant NET release to occur. AsPC-1 cells were also incubated without PMNs to determine if AsPC-1 cells produced any exDNA. Sytox Green staining of AsPC-1 cells confirmed that the increase in exDNA was due to PMNs, as AsPC-1 cells did not carry any exDNA.

### 2.2. AsPC-1-Induced NETs Are Not Contact-Dependent

**Figure 2:**
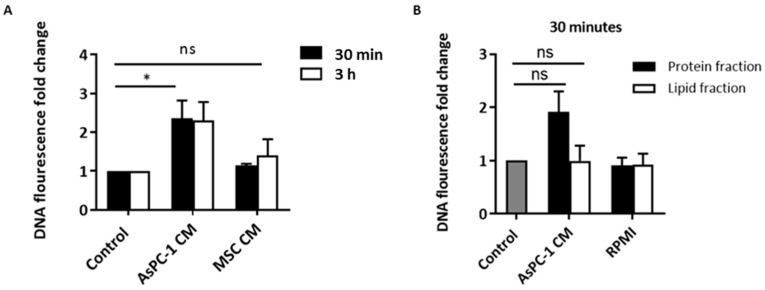
AsPC-1 stimulates NET release via soluble protein mediators. (**A**) The conditioned media (CM) of AsPC-1 cells stimulated the release of NETs. Mesenchymal stem cell (MSC) CM, which served as a control for human non-malignant cells, did not stimulate NET release (*n* = 3, * *p* < 0.01 and ns = non-significant.); (**B**) AsPC-1 CM was separated into lipid and protein fractions, and each was incubated with neutrophils for 30 min. The protein fraction of AsPC-1 CM stimulated NET release while the lipid fraction did not. Media RPMI fractions served as control (*n* = 3). Control: unstimulated neutrophils. One-way ANOVA followed by Bonferroni post-test. Data presented as mean ± SEM.

**Figure 3:**
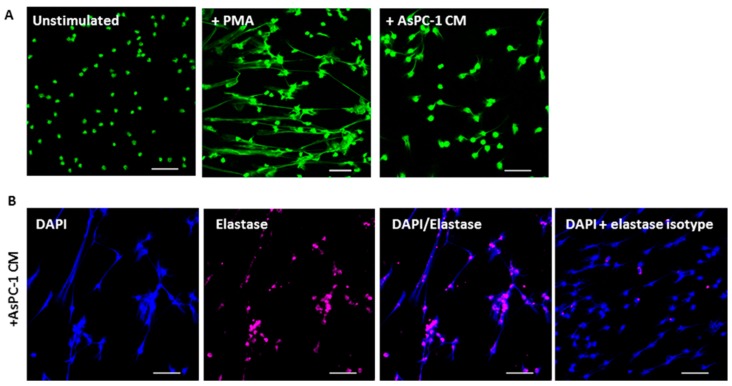
Confocal microscopy images of NETs induced by AsPC-1 conditioned media. (**A**) Neutrophils were incubated on poly-l-lysine coated glass slides and left either unstimulated, or were stimulated with PMA for 3 h, or AsPC-1 CM for 30 min. Neutrophils were fixed with paraformaldehyde and stained with Sytox (green) to visualise extracellular DNA. Images representative of three independent experiments; (**B**) AsPC-1 stimulated neutrophils were also stained with anti-elastase (pink) to visualise elastase bound on NETs. DAPI (blue) was also used as an alternative to visualise DNA. Elastase staining was above isotype control. Images representative of two independent experiments. Scale bar = 50 μm.

**Figure A1:**
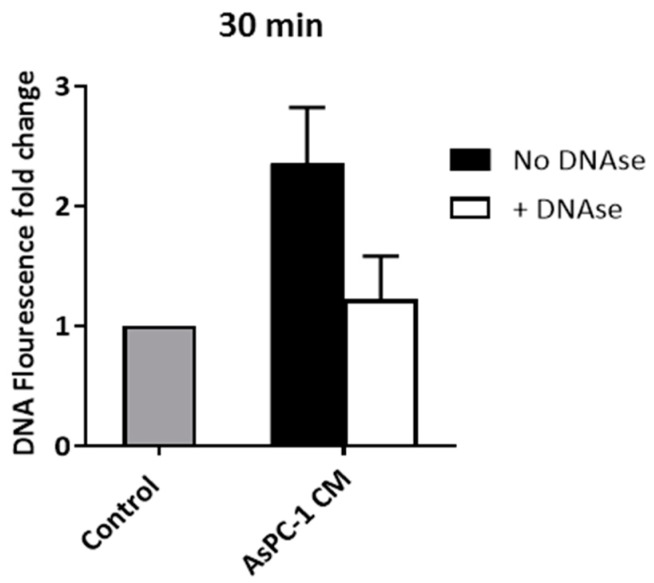
The addition of DNAse I (60 U/mL) abolished extracellular DNA fluorescence to unstimulated control, confirming the specificity of Sytox Green to extracellular DNA (exDNA). Control: unstimulated neutrophils. Data expressed as mean ± SEM from *n* = 3.

To determine whether AsPC-1-induced NETs were mediated through cell–cell contact, or via soluble mediators from AsPC-1, NETs generated were examined after incubation of PMNs with conditioned medium from AsPC-1. Similarly, AsPC-1-conditioned medium (AsPC-1 CM) also resulted in a rapid 2-fold increase in exDNA compared to unstimulated control at 30 min, with no further increase at 3 h (Figure 2). To confirm that the AsPC-1 CM induced NETs phenomenon is specific to cancer cells, the conditioned medium of primary mesenchymal stem cells (MSC) served as a human non-malignant control. Indeed, MSC-conditioned medium did not cause PMNs to release NETs (Figure 2). Fluorescent microscopy confirmed the release of NETs, as a stringy-like expulsion of exDNA can be visualised in AsPC-1 CM stimulated PMNs (Figure 3). At 3 h, PMA-stimulated PMNs show a greater degree of web-like NETs due to PMA being a potent NET activator (Figure 3). To determine if other NET components are present on AsPC-1 CM-induced NETs, neutrophil elastase was also stained. Neutrophil elastase can also be seen extruded on NETs after incubation of PMN with AsPC-1 CM (Figure 3). AsPC-1 CM stimulated PMNs were pre-treated with DNAse I, which resulted in significant decrease in exDNA fluorescence (Figure A1), confirming the specificity of Sytox Green for exDNA.

Furthermore, to determine the nature of the NET-inducing factor/s, AsPC-1 CM was separated into protein and lipid fractions. As the optimal time for NET release induced by AsPC-1 was observed at 30 min, the lipid and protein fractions of AsPC-1 conditioned media were incubated with PMN for 30 min before measuring NET release. Interestingly, the protein fraction of conditioned media resulted in almost 2-fold NET release, while the lipid fraction-stimulated PMNs did not (Figure 2), suggesting that AsPC-1-induced NETs are mediated by soluble proteins and do not rely on direct contact.

### 2.3. AsPC-1-Induced NETs Do Not Require Reactive Oxygen Species Production

**Figure 4:**
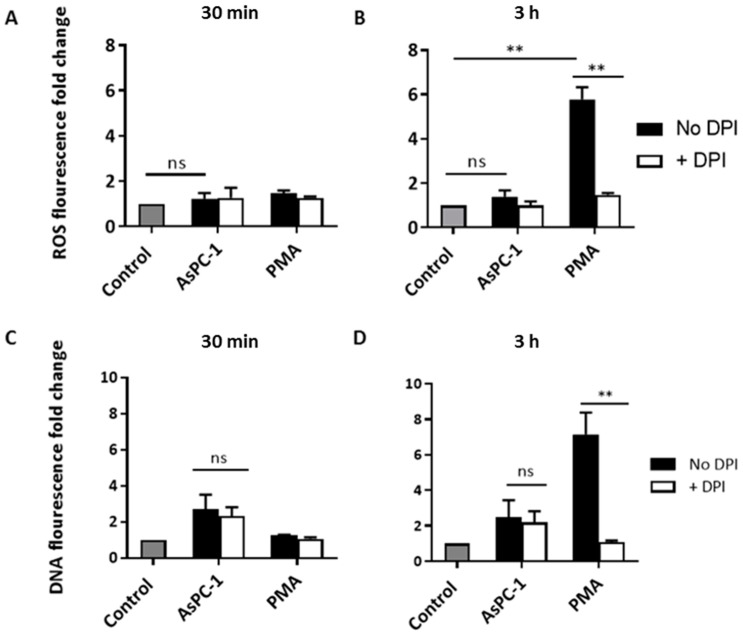
AsPC-1-induced-NET generation is independent of reactive oxygen species production. (**A**,**B**) Quantification of reactive oxygen species (ROS) generated during incubation with AsPC-1 CM and PMA at (**A**) 30 min and (**B**) 3 h using 2′,7′-dichlorodihydrofluorescein diacetate (H2DCFDA) fluorescent dye. ROS were not significantly generated in AsPC-1 CM-stimulated neutrophils at either time points; (**B**) PMA-stimulated neutrophils had significant ROS generated at 3 h, this was abolished with the addition of non-specific ROS inhibitor diphenyleneiodonium (DPI); (**C**,**D**) The effect of DPI on NET release in AsPC-1 or PMA-induced NETs; (**C**) DPI did not reduce AsPC-1-induced NETs; (**D**) NETs were significantly reduced in PMA-stimulated neutrophils by DPI. *n* = 3, ** *p* < 0.001, ns = non-significant. One-way ANOVA followed by Bonferroni post-test. Data presented as mean ± SEM.

Although the mechanism of the generation of NETs is poorly understood, several studies have established that the generation of reactive oxygen species (ROS) is required for the release of NETs [1,22,23]. To examine whether ROS production precedes NET generation in PMNs stimulated by AsPC-1, levels of ROS were measured during PMN incubation with AsPC-1 CM using 2′,7′-dichlorodihydrofluorescein diacetate (DCFDA) indicator dye. Surprisingly, ROS were not detectable in PMNs stimulated with AsPC-1 CM during the 30-min incubation period (Figure 4), the time at which significant AsPC1-induced NETs were observed, while PMA caused a significant accumulation in ROS at 3 h (Figure 4), suggesting that ROS generation is not required for AsPC-1-induced NET. To confirm the ROS-independent release of NETs, a ROS inhibitor, diphenyleneiodonium (DPI), was used to pre-treat PMNs before incubation with AsPC-1 CM. Indeed, AsPC-1 NET release was not affected as exDNA remained unchanged after pre-treatment with DPI (Figure 4). On the other hand, NETs, as well as ROS generation, were significantly reduced to baseline levels in PMA-stimulated PMNs that were pre-treated with DPI (Figure 4).

### 2.4. Platelets Primed by AsPC-1 Stimulate NET Release

**Figure 5:**
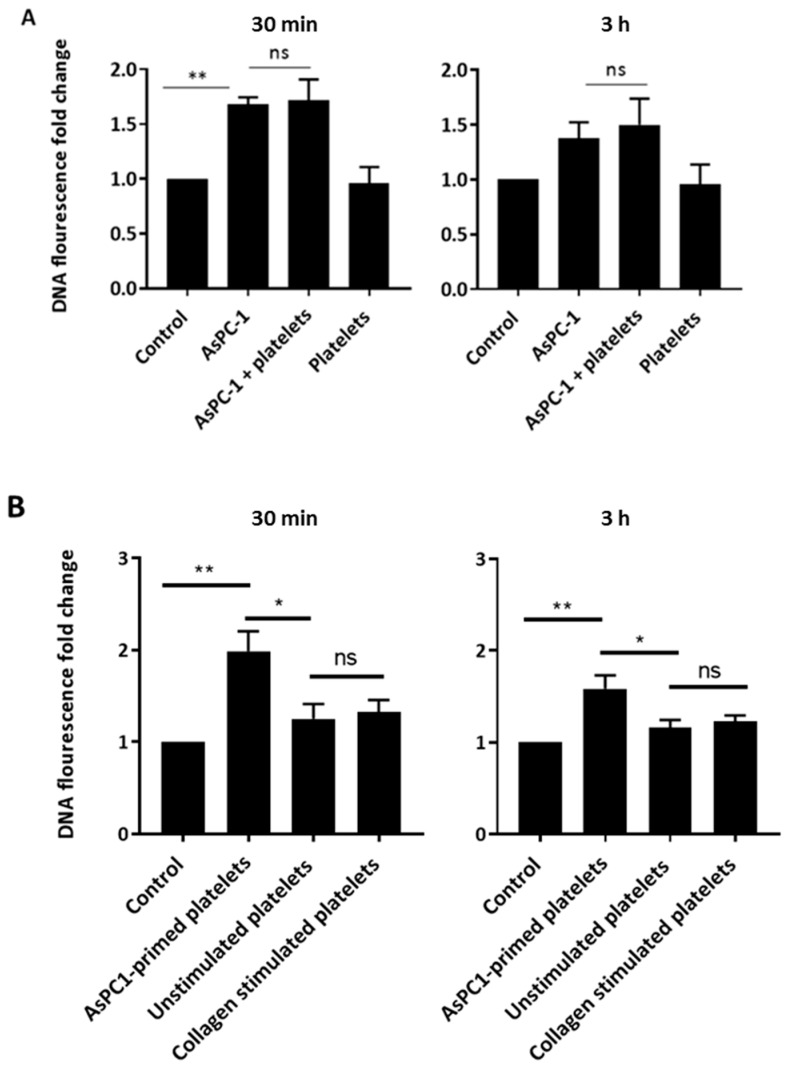
AsPC-1-primed platelets, but not washed platelets, stimulate rapid release of NETs. (**A**) Platelets (1 × 10^8^/mL) were incubated with neutrophils and AsPC-1 cells. Platelets had no effect on AsPC-1-induced NETS; (**B**) the effect of AsPC-1-primed platelets on NET release. Platelets were primed with AsPC-1 cells by incubation with AsPC-1 for 4 h. The supernatant was removed and centrifuged to pellet platelets which were then resuspended and incubated with neutrophils. Primed platelets caused rapid NET release in 30 min. (*n* = 4, * *p* < 0.01, ** *p* < 0.001 and ns = non-significant. One-way ANOVA followed by Bonferroni post-hoc). Control: unstimulated neutrophils. Data expressed as mean ± SEM.

To determine whether platelets play a role in AsPC-1-induced NETs and could exacerbate the production of NETs, platelets were added to the PMN and AsPC-1 co-culture, and NET release was assessed. Platelets did not exacerbate NETs generated by AsPC-1, and unstimulated platelets did not cause NET release which was as expected in the context of normal physiological setting (Figure 5).

Since the addition of platelets to the co-culture did not result in a further increase in NETs, we sought to determine if platelets needed pre-activation or priming with AsPC-1 before co-culturing with PMNs to have any effect on NETs formed. Hence, platelets were pre-incubated with AsPC-1 cells for 4 h before incubating with PMNs. Interestingly, platelets that were primed with AsPC-1 cells caused PMNs to release NETs compared to unstimulated platelets (Figure 5). Furthermore, it was a rapid process that occurred in 30 min (Figure 5). On the other hand, collagen-stimulated platelets did not stimulate increased NET release (Figure 5).

### 2.5. NETs Promote Static Platelet Adhesion and Activation

**Figure 6:**
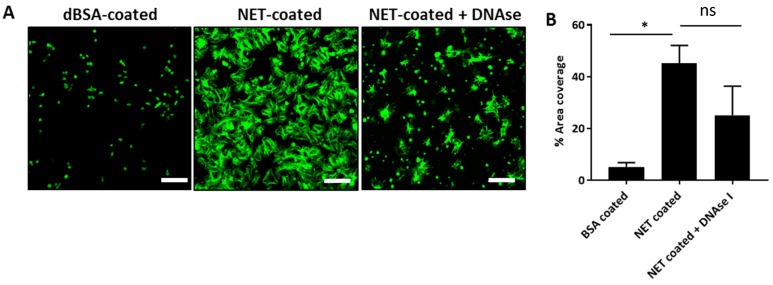
NETs promote static platelet adhesion and activation. (**A**) NETs were isolated from PMA-stimulated neutrophils before coated on glass slides. Washed platelets (2 × 10^7^/mL) were incubated with NETs, and platelet adhesion and spreading were assessed using confocal microscopy. Denatured bovine serum albumin (dBSA)-coated slides served as negative control. NETs caused platelets to adhere and spread. The pre-treatment with deoxyribonuclease I (DNAse I) did not completely abrogate adhesion and spreading. Images representative of three independent experiments. Scale bar = 20 μm; (**B**) quantification of percentage area coverage of platelet adhesion and spreading using Image J analysis software. *n* = 3,* *p* < 0.05 and ns = non-significant. One-way ANOVA followed by Bonferroni post-hoc. Data presented as mean ± SEM.

NETs have been shown to play a role in promoting thrombosis through studies in animal models with venous thrombosis and through platelet and whole blood perfusion assays on NETs [24,25,26]. However, whether NETs are able to directly promote platelet adhesion and activate platelets in static conditions has not been shown. To examine if NETs can directly promote the adhesion and activation of platelets, NETs were isolated from PMA-stimulated PMNs and coated on glass slides for platelet spreading assays. Platelets were then placed on a NET-coated slide and incubated for 1 h before staining the F-actin component of platelets with phalloidin-488, to assess the degree of adhesion and spreading of individual platelets. NETs caused significant adhesion and spreading of platelets as visualised by large spreading of F-actin of platelets across the NET-coated surface, compared to denatured-bovine serum albumin (BSA) coated surface, which served as negative control (Figure 6). Quantification of surface area coverage of platelet spreading also showed a significant increase in spreading on NET-coated slides (Figure 6).

To further investigate which NET component was responsible for platelet adhesion and spreading, NET-coated slides were pre-treated with DNAse I before incubation with platelets. Although to a much lesser extent, platelets were still visualised adhering and spreading on NETs that were pre-treated with DNAse I (Figure 6). Analysis on quantification also found no significant decrease in surface area coverage with DNAse I (Figure 6), suggesting that the protein component in NETs are also capable of promoting platelet adhesion, activation and shape-change.

### 2.6. NETs Are a Scaffold for Dynamic Platelet Adhesion and Thrombus Formation

**Figure 7:**
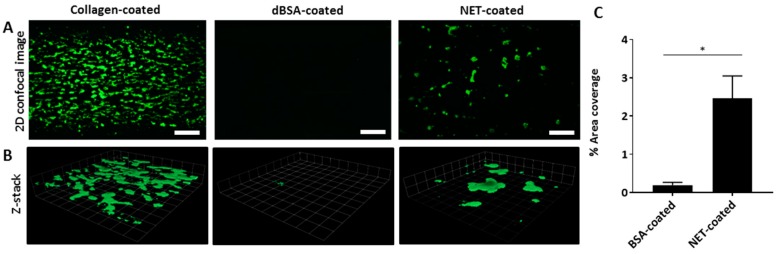
NETs entrap and activate platelets and promote thrombus formation. (**A**) 2D confocal images of platelet adhesion and thrombus formation in collagen-, dBSA-, and NET-coated channels after perfusing DiO6C(3) fluorescently-labelled whole blood for 10 min at 10 dyne/cm^2^. Platelet adhesion and thrombi are visualised in NET-coated channels. Collagen- and dBSA-coated channels served as positive and negative controls, respectively. Scale bar = 90 μm; (**B**) *Z*-stack images confirm the presence of thrombi on NETs, which are similar to those in collagen-coated channels; (**C**) Quantification of surface area coverage of platelet adhesion showed a significant increase in NET-coated channels. *n* = 3, * *p* < 0.05 unpaired *t*-test.

We next investigated the ability of platelets to adhere and activate on NETs and assessed thrombus formation under dynamic conditions. Since venous thrombosis is a common complication in pancreatic cancer patients, we conducted a whole blood perfusion assay over an NET-coated biochip with a venous shear stress of 10 dyne/cm^2^. Whole blood labelled with fluorescent dye DiOC6(3), was perfused over NET-coated channels for 10 min. Platelets were visualised as adhering, and thrombi were formed on NETs (Figure 7, Video S1, available online: https://www.dropbox.com/sh/qlmgmxptzqf4klj/AAA21i7NSjosAG9_zRDPSFxda?dl=0). A greater degree of platelet adhesion and thrombi were formed in collagen-coated channels (positive control), while denatured BSA-coated channels had negligible platelet adhesion (Figure 7; Videos S2 and S3 online). In addition, a significant increase in surface area coverage by platelets was found in NET-coated channels compared to BSA-coated channels (Figure 7). *Z*-stack images also showed 3D visualisation of thrombi that had formed on NETs, similar to that present in collagen-coated channels, while thrombi were absent in BSA-coated channels (Figure 7). Therefore, these data suggest that NETs are able to trap and activate platelets under venous shear stress, and consequently promote thrombus formation.

## 3. Discussion

Pancreatic cancer (PaCa) is associated with high incidence of venous thromboembolism (VTE) [27,28,29], which suggests a close interplay between PaCa cells and platelets, the latter being a key player in haemostasis and thrombosis [30,31]. VTE in cancer is associated with a low survival rate [32]. The high risk of VTE in PaCa patients is mainly associated with a generation of an intrinsic hypercoagulable state [33]. Factors such as tumour-cell induced platelet aggregation, and increased expression of procoagulant factors including tissue factor and thrombin, promote a prothrombotic state which ultimately contributes to the development of VTE. In addition, platelet-neutrophil interactions can coordinate VTE and various pathological conditions [26,34]. In the context of bacterial infection and inflammation, activated platelets can induce NETs [14,35]. It has been known for years that platelet activation is amplified in cancer [36]. In this study, we examined the interplay between platelets, neutrophils and pancreatic cancer cells with a main focus on their impact on NET generation.

### 3.1. AsPC-1 Cells Can Induce NET Release

**Figure A2:**
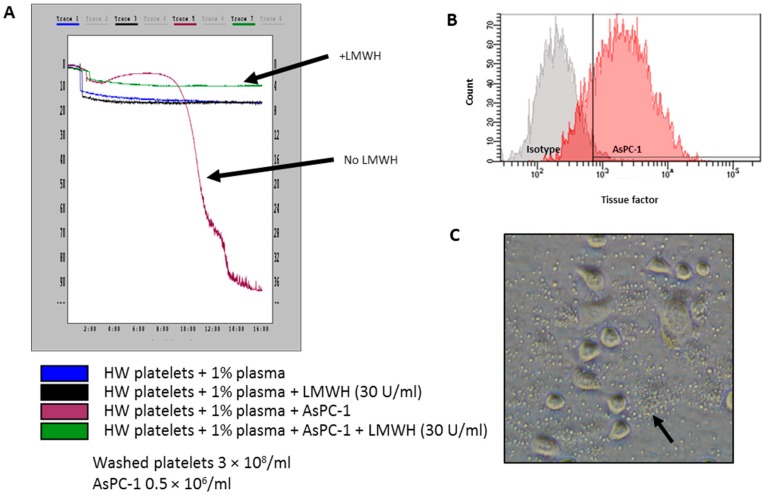
AsPC-1 expresses tissue factor, and causes platelet aggregation (**A**) Light transmission aggregometry showed that AsPC-1-induced aggregation of human washed (HW) platelets, which was tissue factor-dependent, as addition of low-molecular-weight heparin (LMWH) prevented platelet aggregation. The downward-slope represents light transmission approaching 100%, which is indicative of platelet aggregation; (**B**) Flow cytometry analysis of AsPC-1 cells stained with anti-tissue factor show AsPC-1 expressing tissue factor above isotype control; (**C**) Phase-contrast images show platelets aggregating (black arrow) around AsPC-1 cells.

We first examined if pancreatic cancer cells can induce NET release. Here, we show that PaCa AsPC-1 cells can stimulate PMNs to release NETs through direct contact and AsPC-1-derived soluble proteins. Furthermore, we demonstrate that AsPC-1-induced NETs is a cancer specific phenomenon that occurs rapidly and independently of ROS. We have previously shown that AsPC-1 cells in vitro exhibit features that promote coagulation, such as tissue factor expression, and tissue factor-dependent platelet aggregation (Figure A2), which can subsequently contribute to the development of VTE. As NETs have been shown to promote thrombosis, in this study and by others, we propose that NETs that are formed in response to PaCa cells may serve as an additional and novel contributor to the development of VTE.

The release of NETs has been well-established to be a slow process requiring at least 3 to 4 h, however few studies have reported a quick release of NETs occurring within the first hour [14,37]. Similarly, the generation of ROS is thought to be indispensable for forming NETs; however, a few studies have also reported ROS-independent release of NETs [37,38,39]. The majority of NET-inducers studied thus far, stimulate a NET-releasing mechanism that requires at least 3 h of stimulation and is dependent on the generation of ROS [1,22,23]. Herein, we describe a novel mechanism induced by AsPC-1, that is both rapid and ROS-independent. To date, one other study has described a NET mechanism that exhibited both rapid and ROS-independent mechanism of NET release, which was induced by the Gram-positive bacteria *Staphylococcus aureus* [37]. Furthermore, Boone, et al. [40] have recently studied NETs in PaCa in vivo, and reported that NETs released in PaCa are mediated through autophagy pathways. Specifically, the receptor for advanced glycation end products (RAGE) which mediates autophagy in PaCa was found to be necessary for NET release, as neutrophils from RAGE knockout mice were less prone to release NETs. Thus, although we found that PaCa-induced NETs did not depend on ROS generation, they may alternatively be dependent on autophagy which will need to be investigated in future studies.

In addition to an early and ROS-independent release of NETs, we have demonstrated that AsPC-1-induced NETs can be mediated by AsPC-1-derived soluble proteins. Potential mediators may be inflammatory cytokines that are known to be increased in the serum of PaCa patients, and are also released from PaCa cells such as interleukin (IL)-1β, IL-6, IL-8, tumour necrosis factor-alpha (TNF-α), and transforming growth factor-β (TGF-β) [41]. The inflammatory cytokines, TNF-α, IL-8 and IL-1β, have been previously shown to stimulate NET release [42]. Thus, these cytokines may collectively contribute to the activation of PMN and release of NETs; however, further studies are required to delineate the specific mediators. Other cytokines, particularly TGF-β may have a role in polarising neutrophils to a pro-tumourigenic phenotype [43]. Recent studies suggest that neutrophils display plasticity in the tumour microenvironment and can polarise from an N1 antitumour phenotype to an N2 protumour phenotype by TGF-β [44]. Whether or not NET generation is a function of N2 protumour, and not N1 anti-tumour neutrophils, remains to be elucidated.

### 3.2. AsPC-1 Primed Platelets Favour NET Release Compared to Unstimulated Platelets

Neutrophil and platelet interactions are known to exist and play a role in infection and VTE [24]. Likewise, several studies have shown a major role of platelets in mediating interactions or releasing mediators to stimulate NET release [14,15,35,45]. Most studies were carried out in the context of bacterial infection. One study reported that lipopolysaccharide (LPS)-activated platelets were found to mediate NETs, as inhibition of the LPS receptor significantly reduced platelet activation and NET release [14]. Similarly, we observed a trend of increased NET generation when stimulated by platelets that were primed with AsPC-1 cells (or AsPC-1-conditioned media, data not shown), compared to unstimulated platelets. This was not observed when resting platelets were added to the PMN, suggesting that the platelets required priming or pre-activation with AsPC-1 before they could have any effect on PMN activation and NET release. As we have shown that AsPC-1-derived soluble proteins can stimulate NET release, this suggests the potential role of exosomes (small vesicles containing intracellular proteins and RNA) in mediating AsPC-1-induced NETs. Platelets can become ‘educated’ by tumour cells through the transfer of tumour-associated molecules such as proteins or RNA to platelets [46,47,48]. Subsequently, we speculate that upon encounter with PMNs, tumour-educated platelets may either transfer their contents to PMNs, or directly interact with PMNs to induce NET release. Further studies are needed to confirm our observations, and to elucidate what factors from AsPC-1-primed platelets are involved in NET generation. Future investigations may include: (1) characterising of exosomes isolated from AsPC-1; (2) transcriptomic and proteomic profiling of cancer-primed platelets; and (3) assessing the NET-stimulating capability of platelets isolated from tumour-bearing mice.

On the other hand, NETs were not expected to be generated by unstimulated platelets as neutrophil and platelets are constantly in contact within the systemic circulation, and spontaneous NET generation would be undesirable, as it could potentially damage endothelial cells [49] and promote unnecessary coagulation. Similarly, as collagen and platelets are known to play a role in wound healing [50], excessive generation of NETs at a wound site due to collagen-activated platelets is also undesirable, as it may hinder the wound healing process. However, Maugeri, et al. [51] showed that collagen-activated platelets were able to induce NET release. The varying results may be due to different NET quantification methods or number of neutrophils used for stimulation. Maugeri et al. [51] measured soluble DNA to quantify NETs which may have been a more sensitive detection method than the one used here, and the authors also used 10-fold more neutrophils than in our study. NETs generated at wound sites may cause a negative impact and become obstructive as new matrix is deposited. Indeed, a study investigating the role of NETs in a diabetic mouse model, known to have impaired wound healing, found excessive NETs being formed compared to normal mice. Furthermore, degradation of NETs in these diabetic mouse models led to improvements in wound healing [52]. Thus, the generation of NETs by activated platelets may be a regulated process which is favoured in severe conditions where the immune response is compromised, such as in cancer or bacterial infection.

### 3.3. NETs Activate Platelet Dynamics

We have shown for the first time that NETs can directly promote the adhesion, activation and shape change of platelets in static conditions, which corroborates previous findings that NETs are a scaffold for platelet adhesion and aggregation under dynamic conditions [24,25]. The adhesion and activation of platelets was not entirely due to the DNA component of NETs, as the pre-treatment of NETs with DNAse I did not completely abolish platelet adhesion and spreading. This suggests that the protein component of NETs also contributes to platelet adhesion and activation. Histones are the most abundant protein found on NETs [53], and therefore could possibly exert the greatest impact on platelet activation amongst other NET proteins. Extracellular histones have been shown to activate platelets, which in turn promote the generation of plasma thrombin and result in a procoagulant phenotype [54]. Cathepsin G is also present on NETs and has been reported to promote platelet aggregation in vitro [55]. The possible role of these proteins in promoting platelet activation will need to be confirmed by re-assessing platelet adhesion, activation and spreading under the presence of cathepsin G and histone inhibitors, or inhibitors that prevent the mechanism of histone-mediated platelet activation.

We further extended our findings of platelet activation by NETs, and confirmed previous reports by showing that thrombi can form on NETs under dynamic conditions. Our whole blood perfusion assay over NETs was carried out under venous shear conditions, which implicates NETs as a scaffold that can trap platelets and promote thrombus formation within the venous circulation, further corroborating NETs as a mechanism that can contribute to VTE development. We did not investigate the effects of DNAse I on the formation of thrombi under dynamic conditions; however, it will be necessary to determine if DNAse I can serve as a viable drug to reduce VTE risks in pancreatic cancer patients by dissolution of NETs, as we have shown that NETs can be stimulated by pancreatic cancer cells, and can promote venous thrombosis. In addition, heparin may also provide some benefits as it is known to prevent interactions between histones and platelets [56]. As suggested earlier, NET-bound histones may play a major role in platelet activation and subsequent thrombi formation when released in the context of cancer. Thus, heparin may provide additional benefits by reducing or preventing cancer-associated thrombosis.

## 4. Materials and Methods

### 4.1. Neutrophil Isolation

**Figure A3:**
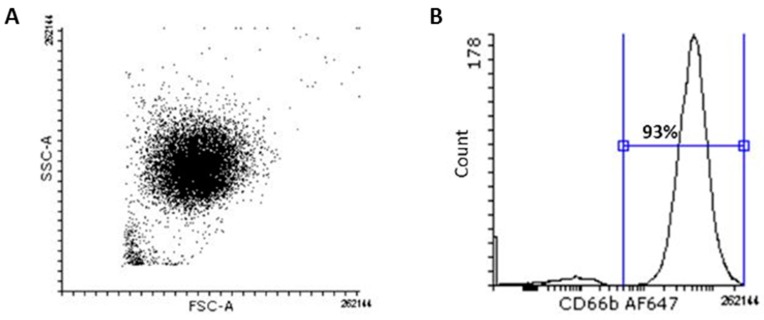
Flow cytometry analysis of CD66b staining on isolated human neutrophils. (**A**) Forward-side-scatter profile of isolated polymorphonuclear neutrophils (PMNs); (**B**) Isolated PMNs were stained with CD66b, a marker for PMNs, to determine purity of neutrophil isolation. A total of 93% of the isolated cell population was positive for CD66b.

A qualified phlebotomist under approved Curtin University Human Research Ethics Committee number HR54/2014 collected blood from healthy volunteers using EDTA (5 mM) as an anticoagulant. Neutrophils were isolated by density gradient centrifugation using PolymorphPrep (Axis-Shield, Oslo, Norway) with minor changes to the manufacturer’s protocol. Briefly, blood was layered over PolymorphPrep then centrifuged at room temperature for 35 min at 600× *g*. The layer containing neutrophils was collected and washed twice at 4 °C in Hank’s buffered saline solution (without calcium and magnesium). Contaminating red blood cells were lysed using Red Blood Cell Lysis buffer (Sigma, St. Louis, MO, USA). Neutrophils were resuspended in ice-cold X-VIVO 15 media (Lonza, Basel, Switzerland) and kept on ice until ready for use in experiments. Cell viability was routinely >99% as assessed by Trypan Blue exclusion. Cell purity was >93% as determined with a haematology analyser (Mindray, Shenzhen, China; BC-VET2800) and flow cytometry staining for Cluster of Differentiation 66b (CD66b) (Figure A3).

### 4.2. Platelet Isolation

Platelets were isolated using the centrifugation method as previously described [57]. Briefly, whole blood anticoagulated with acid citrate dextrose (ACD) was drawn from healthy volunteers then centrifuged at room temperature for 20 min at 150× *g* to obtain platelet-rich plasma (PRP). PRP was collected and platelets pelleted by centrifugation at room temperature for 10 min at 800× *g*. The platelet pellet was washed three times in Citrate-Glucose-Sodium (CGS) buffer (123 mM NaCl, 33.3 mM glucose, 14.7 mM trisodium citrate, pH 7.0) before resuspending in HEPES Tyrode’s buffer (5 mM HEPES (4-(2-hydroxyethyl)-1-piperazineethanesulfonic acid), 5.5 mM glucose, 138 mM NaCl, 12 mM NaHCO_3_, 1.0 mM MgCl_2_·6H_2_O, 2.6 mM KCl, 0.36 mM NaH_2_PO_4_, pH 7.4) supplemented with 1.8 mM CaCl_2_ for use in experiments. Prostaglandin I_2_ (PGI_2_; 0.5 μM) was added to whole blood and each wash to minimise platelet activation during the preparation process.

### 4.3. NET Quantification Assay

Neutrophils (5 × 10^4^) were seeded in 96-well clear-bottom plate and allowed to adhere for 30 min at 37 °C 5% CO_2_ before adding treatments, phorbol myristate acetate (PMA, 25 nM), AsPC-1 cells (10 × 10^4^), collagen (4 μg/mL), platelets (1 × 10^8^/mL), or replacing the supernatant with AsPC-1 conditioned media. After 30 min incubation with treatment, NET release was quantified by staining extracellular DNA with cell-impermeable Sytox^®^ Green (5 μM; ThermoFisher, Waltham, MA, USA) and measurement of fluorescence emission at 523 nm (488 nm laser excitation). Fluorescent measurement was performed with EnSpire Multimode Plate Reader (PerkinElmer, Waltham, MA, USA). A second reading was obtained at 3 h. Treatments were performed in replicates of three. The amount of NET released was determined by calculating fluorescence fold change from unstimulated control.

### 4.4. Fluorescence Microscopy and Immunofluorescence

Neutrophils (1 × 10^6^/mL) were seeded on poly-L-lysine-coated glass slides then incubated with AsPC-1 conditioned media for 30 min, or PMA (25 nM) for 3 h at 37 °C. Extracellular DNA was stained with cell-impermeable Sytox^®^ Green (2 μM) before fixing the cells in 100% ice-cold methanol for 5 min at room temperature. Samples were blocked in 10% goat serum for 30 min at room temperature, then incubated with rabbit anti-elastase (1 μg/mL; Abcam #68672, Cambridge, UK) in 1% bovine serum albumin (BSA) overnight at 4 °C. Rabbit IgG (Cell Signaling Technology, Danvers, MA, USA) at the same concentration was used as an isotype control for anti-elastase antibody. Cells were washed three times in phosphate-buffered saline (PBS) before incubating with anti-rabbit Alexa594-conjugated secondary antibody (1/500; Cell Signalling). Samples were mounted with ProLong Gold mounting media with DAPI (Molecular Probes, Life Technologies) and imaged using Nikon A1+ confocal microscope.

### 4.5. Reactive Oxygen Species Quantification

Neutrophils (5 × 10^4^) were seeded in 96-well black-bottom plate and allowed to incubate for 30 min in 37 °C 5% CO_2_, before pre-treating with a cell-permeant 2′,7′-dichlorodihydrofluorescein diacetate (H_2_DCFDA, 10 μM; Sigma-Aldrich, St. Louis, MO, USA) for 15 min. H_2_DCFDA is a non-fluorescent dye that is cleaved by intracellular esterases to a membrane-impermeable H_2_DCF, which can subsequently react with a variety of reactive oxygen species to emit a highly fluorescent 2′,7′-dichlorofluorescein (DCF) [58]. Excess dye was removed and replaced with X-VIVO 15 media (Lonza, Basel, Switzerland). Neutrophils were then either left untreated or pre-treated for 15 min with ROS inhibitor diphenyleneiodonium (DPI; 1 μM). Neutrophils were finally treated with stimulants before measuring ROS fluorescence on an Enspire Multimode Plate Reader (PerkinElmer, Waltham, MA, USA) at 523-nm fluorescence emission with 488-laser excitation. The quantity of ROS generated in treated neutrophils was determined by calculated fluorescence fold change from unstimulated control.

### 4.6. Preparation of Conditioned Media

AsPC-1 cells (purchased from American Type Culture Collection, Mannassasa, VA, USA) were grown in RPMI-1640 medium (ThermoFisher, Waltham, MA, USA) supplemented with 2 mM l-glutamine, 10 mM HEPES (HyClone, ThermoFisher) 1 mM sodium pyruvate (Gibco, ThermoFisher), 4500 mg/L glucose, and 500 mg/L sodium bicarbonate and 10% fetal bovine serum (Bovogen, Victoria, Australia), in an incubator maintained at 37 °C and 5% CO_2_. At 80%–90% confluence, the medium was changed to phenol red-free RPMI-1640 without FBS, and cells were cultured for a further 36 h. The supernatant (conditioned medium, CM) was collected and centrifuged at 12,000× *g* for 10 min at 4 °C to remove cell debris. CM was aliquoted and stored at −80 °C. Human primary mesenchymal stem cells (MSC), obtained from Lonza (Basel, Switzerland), were similarly cultured in RPMI-1640 complete medium. Conditioned medium from MSC were prepared as above. Both cell lines were negative for mycoplasma throughout all the studies (routine testing by Curtin Health Innovation Research Institute, Perth, Australia).

### 4.7. Protein and Lipid Fractionation of Conditioned Media

The protein and lipid fractions were separated as described previously with slight modifications [59]. Briefly, conditioned media were mixed with 100% butanol (1:2) and vortexed before incubating at 4 °C for 90 min. After incubation, protein and lipid phases were separated by centrifugation at 1000× *g* for 10 min. The lipid (upper) and protein (lower) phases were separated and dried using a SpeedVac system (Eppendorf, Concentrator 5301, Hamburg, Germany) at 30 °C for 7 h. The dry pellet was resuspended in PBS to 1/5 of the starting volume.

### 4.8. Cell-Free NET Isolation

NETs were isolated as previously described [60]. Briefly, neutrophils (5 × 10^6^/mL) were incubated with 600 nM PMA for 4 h at 37 °C. The supernatant was removed and NET monolayer detached from the culture surface with ice-cold PBS, then centrifuged at 400× *g* for 5 min at 4 °C to pellet cell debris. The cell-free supernatant was pooled and centrifuged at 15,000× *g* for 15 min at 4 °C to pellet DNA. DNA pellet was resuspended in PBS to a volume corresponding to 100 μL per 1 × 10^7^ neutrophils to obtain cell-free NETs.

### 4.9. Platelet Spreading Assay

Glass slides were coated with 1% denatured BSA (dBSA) in PBS or cell-free NET overnight at 4 °C in a humidifier chamber. NET-coated slides were either left untreated or pre-treated with DNAse I (100 U/mL; Stemcell Technologies, Vancouver, BC, Canada) in Hank’s buffered saline solution (with calcium). The coated slides were then washed with 1× PBS and blocked with 1% dBSA for 1 h at room temperature, in the humidifier chamber. Denatured BSA was prepared by heating the solution in PBS without calcium or magnesium to 80 °C for 3 min, immediately put on ice until cool, and aliquoted and stored at −20 °C until use.

Platelets (2 × 10^7^/mL) were seeded on the coated slides for 1 h at 37 °C CO_2_ in the humidifier chamber. Adherent platelets were washed with PBS before fixing in 4% paraformaldehyde for 10 min at room temperature, permeabilised with 0.1% Triton-X 100 for 2 min, and washed once with PBS. Platelets were then stained with Alexa Flour 488-conjugated phalloidin (diluted 1:100 in PBS, ThermoFisher, Waltham, MA, USA) for 20 min in the dark. Samples were mounted with ProLong Gold antifade reagent (Molecular Probes). Images were taken with Nikon A1+ confocal microscope with 100× objective. Three random fields of view were taken for analysis using Image J software (Version 1.50i, National Institute of Health, Bethesda, MD, USA).

### 4.10. Perfusion Assay—Ex Vivo Thrombus Formation

Microfluidics biochip (Cellix Ltd., Dublin, Ireland) channels were coated with cell-free NETs, 1% dBSA or 100 μg/mL collagen type I overnight at 4 °C and blocked in 1% dBSA for 1 h. Citrated-whole blood (1:9, 3.8% *w*/*v* sodium citrate to whole blood) was drawn from healthy volunteers and fluorescently-labelled with DiOC_6_(3) (5 μM; Molecular Probes) for 15 min in the dark. Blood was perfused through the channels at a venous shear rate of 10 dyne/cm^2^ for 10 min using a Mirus microfluidics pump (Cellix Ltd.), after which the samples were washed at the same rate for 2 min in PBS. Video live imaging of the perfusion and image snaps of formed thrombi were captured using UltraView Vox spinning disk confocal microscope. Four fields of view were taken after each perfusion assay for surface area quantification using Image J (Version 1.50i, National Institute of Health). *Z*-stack images were also acquired.

### 4.11. Statistical Analysis

All statistical analysis was performed on GraphPad Prism 7 (GraphPad Software, Inc., San Diego, CA, USA). Results are shown as mean ± SEM. The one-way ANOVA with post-hoc Bonferroni was performed to determine statistical significance between means of multiple groups. The unpaired *t*-test was used to compare means between two groups. *p* ≤ 0.05 was considered statistically significant.

## 5. Conclusions

Taken together, our results demonstrate that pancreatic cancer cells can stimulate NET formation, and in turn NETs produced are a platform for platelet adhesion and thrombi formation. The degradation of NETs by DNAse I, and/or prevention of histone-platelet interaction by heparin, could become a potential new drug option for pancreatic cancer patients if proven effective to reduce venous thrombosis, and may lead to improved survival rates as VTE is linked to poor prognosis in PaCa patients. Moreover, since NETs have also been implicated in metastasis, targeting NETs may not only reduce risk of developing VTE, but it may also attenuate the development of highly metastatic pancreatic tumours. Thus, it will be important to look at the effects of NETs on pancreatic cancer cell survival, proliferation, migration and invasion, to determine if NETs exhibit pro-tumourigenic characteristics, and serve as another reason to be targeted in pancreatic cancer patients.

## Abbreviations

NETs	Neutrophil Extracellular Traps
VTE	Venous Thromboembolism
PaCa	Pancreatic Cancer
